# Enzamin ameliorates adipose tissue inflammation with impaired adipocytokine expression and insulin resistance in db/db mice

**DOI:** 10.1017/jns.2013.34

**Published:** 2013-12-03

**Authors:** Yukinori Tamura, Masato Yano, Naoyuki Kawao, Katsumi Okumoto, Shigeru Ueshima, Hiroshi Kaji, Osamu Matsuo

**Affiliations:** 1 Department of Physiology and Regenerative Medicine, Kinki University Faculty of Medicine, Osakasayama, Japan; 2 Life Science Research Institute, Kinki University, Osakasayama, Japan; 3 Department of Food Science and Nutrition, Kinki University Faculty of Agriculture, Nara, Japan; 4 Kinki University Faculty of Medicine, Osakasayama, Japan

**Keywords:** Insulin resistance, Adipocytokines, Macrophages, Enzamin, ATCC, American Type Culture Collection, CPT, carnitine palmitoyltransferase, CPT1a, carnitine palmitoyltransferase 1 (liver), CPT1b, carnitine palmitoyltransferase 1 (muscle), CT, computed tomography, LPS, lipopolysaccharide, MCP-1, monocyte chemoattractant protein 1, Nox2, NADPH oxidase 2, PAI-1, plasminogen activator inhibitor 1, t-PA, tissue-type plasminogen activator

## Abstract

The effects of Enzamin on obesity-related metabolic disorders in obese db/db mice were examined to explore a novel agent for the prevention of insulin resistance. Db/db mice were treated with water containing Enzamin (0·1 and 1·0 %) for 8 weeks from 6 weeks of age. Enzamin treatment at 1·0 %, but not at 0·1 %, significantly decreased the fasting plasma glucose, serum total cholesterol and TAG levels in db/db mice, without affecting body weight gain and body fat composition. Furthermore, insulin sensitivity and glucose tolerance were improved by the treatment of db/db mice with 1·0 % Enzamin. Immunohistochemical studies and gene expression analysis showed that 1·0 % Enzamin treatment suppressed macrophage accumulation and inflammation in the adipose tissue. In addition, 1·0 % Enzamin treatment increased serum adiponectin in db/db mice. Treatment with 1·0 % Enzamin also significantly suppressed the expression of NADPH oxidase subunits, suggesting an antioxidative effect for Enzamin in the adipose tissue. Furthermore, *in vitro* experiments demonstrated that the lipopolysaccharide-induced inflammatory reaction was significantly suppressed by Enzamin treatment in macrophages. Enzamin treatment increased the expression of GLUT4 mRNA in muscle, but not GLUT2 mRNA in the liver of db/db mice. Enzamin also increased the mRNA expression of carnitine palmitoyltransferase 1a (CPT1a, muscle isoform) in db/db mice, whereas Enzamin treatment did not affect the mRNA expression of CPT1b (liver isoform) in db/db mice. In conclusion, our data indicate that Enzamin can improve insulin resistance by ameliorating impaired adipocytokine expression, presumably through its anti-inflammatory action, and that Enzamin possesses a potential for preventing the metabolic syndrome.

The metabolic syndrome, which is characterised by a clustering of visceral obesity, impaired glucose tolerance, hypertension and dyslipidaemia, is a major cause of type 2 diabetes and atherothrombosis^(^^1^^)^. Visceral obesity and insulin resistance are thought to represent common underlying factors of the metabolic syndrome^(^^2^^)^. It is critical therefore to develop novel agents for the prevention or treatment of obesity and insulin resistance.

Many reports have indicated that obesity is associated with a state of chronic, low-grade inflammation, suggesting that inflammation may be a potential mechanism whereby obesity can lead to insulin resistance^(^^3^^)^. Indeed, obesity and insulin resistance are strongly associated with systemic markers of inflammation, and inflammation has been recognised clinically as a major predictor of atherosclerotic diseases^(^^4^^)^.

The adipose tissue is an important endocrine organ that secretes numerous biologically active molecules, such as leptin, adiponectin, TNF-α, monocyte chemoattractant protein 1 (MCP-1) and plasminogen activator inhibitor 1 (PAI-1), which are collectively termed adipocytokines^(^^5^^,^^6^^)^. The impaired production of pro-inflammatory and anti-inflammatory adipocytokines seen in visceral fat obesity is associated with the metabolic syndrome^(^^7^^)^, indicating that inflammatory changes in the adipose tissue may contribute to the development of several aspects of the metabolic syndrome and result in type 2 diabetes and thrombotic diseases^(^^8^^)^.

Enzamin is a product from *Bacillus subtilis* AK and *Lactobacillus*, treated under physically adverse conditions such as heat treatment, and acid treatment^(^^9^^)^. We have shown previously that Enzamin displays profibrinolytic anti-thrombotic properties both *in vitro* and *in vivo*^(^^9^^)^. However, the effects of Enzamin on the metabolic syndrome have not yet been elucidated. In the present study, we therefore examined the influence of Enzamin on insulin resistance and adipocytokine expression in obese diabetic db/db mice.

## Experimental methods

### Materials

The following materials were obtained from the commercial sources indicated: human regular insulin (Eli Lilly), anti-F4/80 antibody (AbD Serotec), Glutest Ace (Sanwa Kagaku Kenkyusho), RNeasy mini kits (Qiagen), rat monoclonal anti-F4/80 antibody (AbD Serotec), anti-rat secondary antibodies conjugated with horseradish peroxidase (Cell Signaling Technology Japan), fetal bovine serum (American Type Culture Collection (ATCC)) and lipopolysaccharides (LPS) from *Escherichia coli* 0111:B4 (Sigma Aldrich Japan). All other reagents and chemicals were of the highest grade available. Enzamin, which was produced by the long-term fermentation and self-digestion of *Bacillus subtilis* AK and *Lactobacillus*^(^^10^^)^, was provided by the Enzamin Research Institute, Osaka, Japan. Japan Food Research Laboratories, which issue the official documents for the food export, analysed the molecular distribution of Enzamin by HPLC with a TSKgel G2500PWXL column (TOSOH Corporation). The standard molecular markers were cytochrome *c* (12500 Da), aprotinin (6152 Da), bacitracin (1450 Da), angiotensin II (1046 Da), Gly-Gly-Tyr-Arg (451 Da) and Gly-Gly-Gly (189 Da). There was an abundance of peaks and the majority were below the molecular weight of 500 Da (75 %). There were peaks with the molecular weight of 500–1000 Da (10 %), 1000–3000 Da (11 %), 3000–6000 Da (2 %) and over 6000 Da (2 %), respectively.

### Animal preparation and experimental design

Male db/db mice and db/ + m mice were obtained from Charles River at 5 weeks of age. The db/db mice were fed on normal chow and water *ad libitum* supplemented with 0, 0·1 or 1·0 % Enzamin for 8 weeks from 6 weeks of age. Each 0 % Enzamin group is referred to below as the control. The db/ + m mice were fed on normal chow and water *ad libitum* as lean mice. All animals were maintained on a 12 h light–dark cycle. The animal experiments were conducted according to the Guidelines for Animal Experiments at Kinki University Faculty of Medicine.

### Body fat composition analysis

Body fat composition was estimated by computed tomography (CT) analysis in mice that were anaesthetised with forane (Isoflurane; Abbott Japan) and then scanned using a LaTheta (LCT-200) experimental animal CT system (Hitachi-Aloka Medical)^(^^11^^)^. Contiguous 1-mm slice images between L1 and L5 were used for quantitative assessment employing LaTheta software (version 3·40). Visceral fat and subcutaneous fat were distinguished, and the total fat content, visceral fat weight and subcutaneous fat weight were calculated from all slice images and evaluated quantitatively.

### Analysis of metabolic parameters

The plasma insulin, serum total cholesterol, TAG, adiponectin and TNF-α levels were measured using an insulin assay kit (Morinaga Institute of Biological Science), Cholesterol E test, Triglyceride E test (Wako Pure Chemical Industries), adiponectin ELISA kit (Otsuka Pharmaceutical) and a Quantikine TNF-α ELISA kit (R&D Systems), respectively. For glucose tolerance tests, mice were deprived of food for 16 h and glucose (1·5 g/kg body mass) was then injected intraperitoneally. For insulin tolerance tests, mice were injected intraperitoneally with human regular insulin (10·5 μg/kg body mass for db/+ m mice and 35·0 μg/kg body mass for db/db mice). Blood samples were collected before and after each injection, and the plasma glucose concentration was measured with a Glutest Ace.

### Cell culture

RAW 264.7 (ATCC number: TIB-71), a murine macrophage cell line, was purchased from ATCC and maintained in Dulbecco's modified essential medium supplemented with 10 % fetal bovine serum and 100 mg/ml penicillin–streptomycin at 37°C with 5 % CO_2_. RAW 264.7 cells (1 × 10^6^ cells/well) were plated on six-well cell culture plates and incubated for 24 h. Cells were treated with 0·01 or 0·1 % concentrations of Enzamin in the presence or absence of 1 µg/ml LPS for 12 h. After washing twice with PBS, the total cellular RNA was extracted for gene expression analysis employing real-time PCR.

### Quantitative real-time PCR

Total RNA was extracted from the frozen adipose tissue (100 mg), liver tissue (30 mg) and muscle tissue (30 mg) of mice and from RAW 264.7 cells using an RNeasy mini kit (Qiagen). The cDNA was synthesised from the total RNA using Super Script III (Life Technologies Japan). The real-time PCR was performed on a StepOne Plus using the SYBR® Green PCR Master Mix (Life Technologies Japan). The primer sets are listed in Table 1. The mRNA levels were normalised relative to the amount of 18s ribosomal RNA, and expressed in arbitrary units.

**Table 1.** Primers used for real-time PCR

Primer		Sequence
GLUT2	Forward	5′-GGCTAATTTCAGGACTGGTT-3′
	Reverse	5′-TTTCTTTGCCCTGACTTCCT-3′
GLUT4	Forward	5′-CATGGCTGTCGCTGGTTTC-3′
	Reverse	5′-AAACCCATGCCGACAATGA-3′
G6Pase	Forward	5′-CTGTGAGACCGGACCAGGA-3′
	Reverse	5′-GACCATAACATATACACCTGCTGC-3′
CPT1a	Forward	5′-GTCCCAGCTGTCAAAGATAC-3′
	Reverse	5′-GGAAGTATTGAAGAGTCGC-3′
CPT1b	Forward	5′-CAAGTTCAGAGACGAACGCC-3′
	Reverse	5′-TCAAGAGCTGTTCTCCGAACTG-3′
TNF-α	Forward	5′-CCCAGACCCTCACACTCAGATC-3′
	Reverse	5′-GCCACTCCAGCTGCTCCTC-3′
MCP-1	Forward	5′-CCACTCACCTGCTGCTACTCA-3′
	Reverse	5′-TGGTGATCCTCTTGTAGCTCTCC-3′
IL-6	Forward	5′-GTTCTCTGGGAAATCGTGGA-3′
	Reverse	5′-GGAAATTCGGGGTAGGAAGGA-3′
PAI-1	Forward	5′-TTCAGCCCTTGCTTGCCTC-3′
	Reverse	5′-ACACTTTTACTCCGAAGTCGGT-3′
Emr-1	Forward	5′-CTTTGGCTATGGGCTTCCAGTC-3′
	Reverse	5′-GCAAGGAGGACAGAGTTTATCGTG-3′
CD68	Forward	5′-CTTCCCACAGGCAGCACAG-3′
	Reverse	5′-AATGATGAGAGGCAGCAAGAGG-3′
TLR-4	Forward	5′-TATCCAGGTGTGAAATTGAAACAATT-3′
	Reverse	5′-GGGTTTCCTGTCAGTATCAAGTTTG-3′
Nox2	Forward	5′-TTGGGTCAGCACTGGCTCTG-3′
	Reverse	5′-TGGCGGTGTGCAGTGCTATC-3′
P22^phox^	Forward	5′-GTCCACCATGGAGCGATGTG-3′
	Reverse	5′-CAATGGCCAAGCAGACGGTC-3′
P47^phox^	Forward	5′-GATGTTCCCCATTGAGGCCG-3′
	Reverse	5′-GTTTCAGGTCATCAGGCCGC-3′
18S rRNA	Forward	5′-CGGCTACCACATCCAAGGAA-3′
	Reverse	5′-GCTGGAATTACCGCGGCT-3′

G6Pase, glucose-6-phosphatase; CPT1a, carnitine palmitoyltransferase 1 (liver); CPT1b, carnitine palmitoyltransferase 1 (muscle); MCP-1, monocyte chemoattractant protein 1; PAI-1, plasminogen activator inhibitor 1; Emr-1, EGF-like module-containing mucin-like hormone receptor-like 1; TLR-4, Toll-like receptor 4; Nox2, NADPH oxidase 2; rRNA, ribosomal RNA.

### Histological analysis

Adipose tissue was fixed for 12–16 h at 4°C with 4 % paraformaldehyde, and embedded in paraffin. Sections of 4 µm thickness were incubated with rat monoclonal anti-F4/80 antibody. The sections were then incubated with the appropriate anti-rat secondary antibodies conjugated with horseradish peroxidase. Positive signals were visualised employing a tyramide signal amplification system (PerkinElmer). The sections were counterstained with 4′,6-diamidino-2-phenylindole (DAPI) and photographed using a fluorescence microscope (E800; Canon) with a CCD camera (Keyence Japan). For each individual mouse tissue block, ten fields from each section were analysed. The total number of nuclei and the number of nuclei of F4/80-expressing cells were counted for each field by employing an image processing program (NIH image; National Institutes of Health) in a blinded evaluation. The fraction of F4/80-expressing cells for each section was calculated as the sum of the number of nuclei of F4/80-expressing cells divided by the total number of nuclei in the sections.

### Statistical analysis

All data were expressed as mean values with their standard errors. The statistical significance of differences was assessed by the unpaired *t* test and one-way ANOVA. Differences with *P* < 0·05 were regarded as significant. All statistical analyses were performed using StatView version 5.0 software (SAS Institute Inc.).

## Results

### Effect of Enzamin on body fat composition in db/db mice

**Fig. 1.**
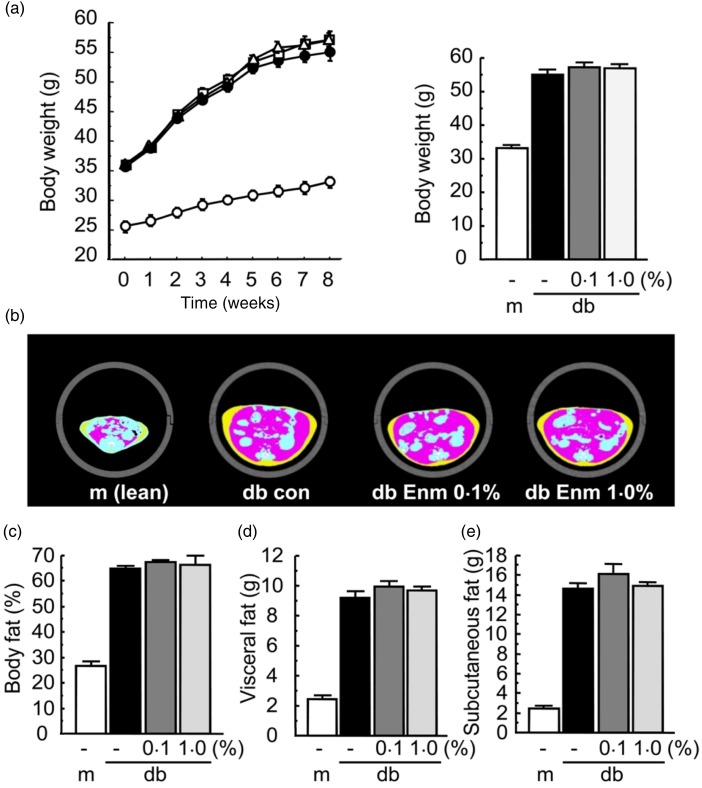
Effect of Enzamin (Enm) treatment for 8 weeks on body weight and fat composition. (a) Growth curves during the experiment (left) and body weight at the end of the experiment (right) of non-treated db/ + m (lean control) and db/db (obese control) mice, and 0·1 and 1·0 % Enzamin-treated db/db mice. ○, db/ + m mice (*n* 6); •, non-treated db/db mice (*n* 10); ∆, 0·1 % Enzamin-treated db/db mice (*n* 9); □, 1·0 % Enzamin-treated db/db mice (*n* 8). (b) Representative computed tomography (CT) sections of abdominal regions. con, Control. Pink areas show visceral fat; yellow areas show subcutaneous fat. (c) Total fat content, and weights of visceral (d) and subcutaneous (e) fat in db/ + m and db/db mice following each treatment, calculated from CT scan data. m, db/ + m; db, db/db. Data are means (*n* 6–10 per group), with standard errors represented by vertical bars.

Treatment with 1 % Enzamin exerted no effect on water intake, whereas treatment with 0·1 % Enzamin increased water intake slightly. Enzamin had no effect on food intake (data not shown) or body weight gain in db/db mice (Fig. 1(a)). To examine the effect of Enzamin treatment on body fat composition, we next performed CT scan analysis. Abdominal CT images at L2 demonstrated that both the visceral and subcutaneous fat areas were markedly increased in db/db mice as compared with db/+ m mice, but that Enzamin treatment exerted no effect on the visceral and subcutaneous fat areas in db/db mice (Fig. 1(b)). The total fat content, visceral fat weight and subcutaneous fat weight calculated from the CT scan analysis were not affected by Enzamin treatment in db/db mice (Fig. 1(c)–(e)). Enzamin treatment also had no effect on the liver fat content estimated by CT scan analysis in db/db mice (data not shown).

### Effect of Enzamin on lipid and glucose metabolism in db/db mice

**Fig. 2.**
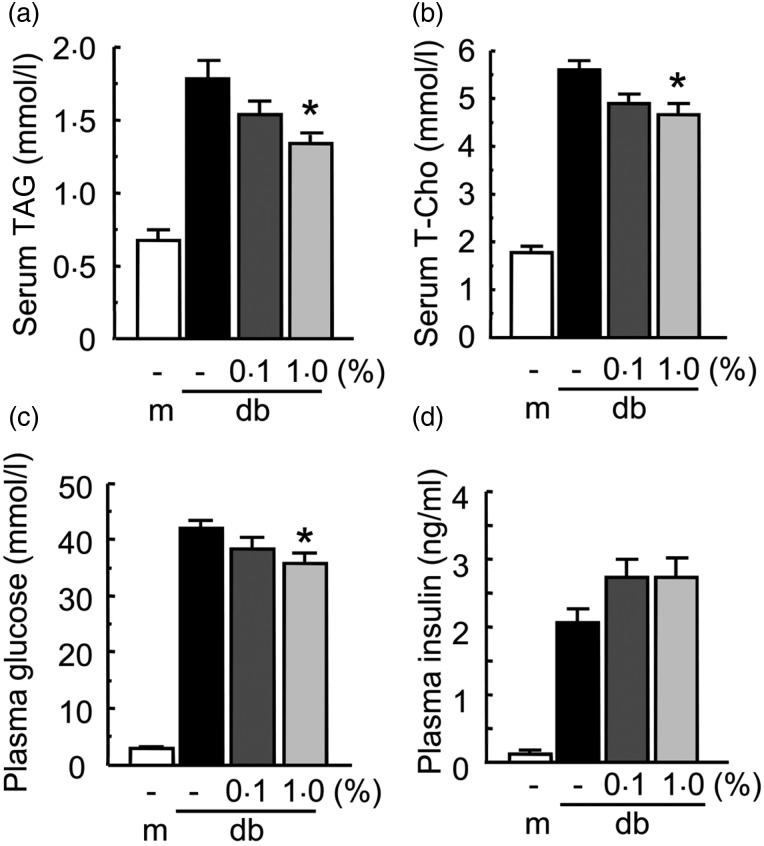
Effect of Enzamin treatment for 8 weeks on lipid and glucose metabolism. Fasting serum TAG (a), total cholesterol (T-Cho) (b), plasma glucose (c) and insulin (d) concentrations in non-treated db/+ m (lean control, *n* 6) and db/db (obese control, *n* 10) mice, and 0·1 % (*n* 9) and 1·0 % (*n* 8) Enzamin-treated db/db mice. m, db/ + m; db, db/db. Data are means (*n* 6–10 per group), with standard errors represented by vertical bars. *Mean value was significantly different from that of the non-treated db/db mice (*P* < 0·05).

To evaluate the effect of Enzamin treatment on lipid metabolism, we measured serum TAG and total cholesterol levels in db/db mice. The fasting serum TAG levels in db/db mice were higher than those in db/+ m mice (Fig. 2(a)). Treatment with Enzamin at 0·1 % slightly reduced the serum TAG level, and 1·0 % Enzamin treatment significantly reduced the fasting serum TAG level in db/db mice (Fig. 2(a)). Similarly, the fasting serum total cholesterol level was slightly but not significantly decreased by 0·1 % Enzamin, and was significantly decreased by 1·0 % Enzamin in db/db mice (Fig. 2(b)). Thus, Enzamin treatment reduced fasting serum TAG and cholesterol levels dose-dependently in db/db mice. Taken together, these findings suggest that Enzamin treatment improves lipid metabolism in db/db mice.

We next evaluated the effect of Enzamin treatment on glucose metabolism in db/db mice. The fasting plasma glucose was markedly increased in db/db mice as compared with db/+ m mice (Fig. 2(c)). Enzamin treatment at 1·0 %, but not at 0·1 %, significantly reduced the fasting plasma glucose level in db/db mice (Fig. 2(c)). Although the fasting plasma insulin was also markedly elevated in db/db mice as compared with db/+ m mice (Fig. 2(d)), there were no differences in fasting plasma insulin levels between non-treated db/db mice and Enzamin-treated db/db mice (Fig. 2(d)).

**Fig. 3.**
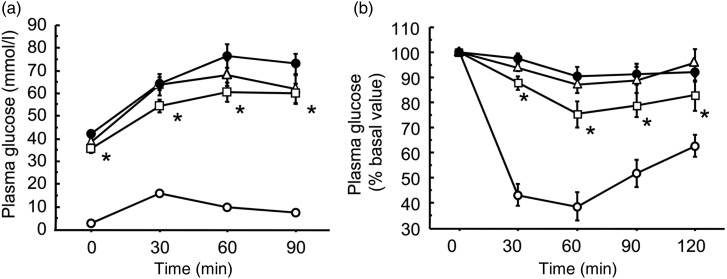
Effects of Enzamin treatment for 8 weeks on glucose tolerance and insulin sensitivity. Responses of plasma glucose to a single intraperitoneal injection of glucose (a) or insulin (b) in non-treated db/ + m (○, *n* 6) and db/db (•, *n* 10) mice, and 0·1 % (∆, *n* 9) and 1·0 % (□, *n* 8) Enzamin-treated db/db mice. m, db/ + m; db, db/db. Data are means (*n* 6–10 per group), with standard errors represented by vertical bars. *Mean value was significantly different from that of the non-treated db/db mice (*P* < 0·05).

To evaluate further the effects of Enzamin on glucose metabolism, we next performed an intraperitoneal glucose tolerance test and intraperitoneal insulin tolerance test. The plasma glucose level after intraperitoneal glucose injection was dramatically elevated in db/db mice as compared with db/+ m mice (Fig. 3(a)), indicating severe glucose intolerance. Enzamin treatment at 1·0 %, but not at 0·1 %, significantly suppressed the elevation of the plasma glucose level in db/db mice at 30 min after glucose injection. Furthermore, a similar suppressive effect was also noted at 60 and 90 min after intraperitoneal glucose injection (Fig. 3(a)). These data suggest that Enzamin can improve glucose intolerance in db/db mice. Severe impairment of insulin sensitivity was also observed in db/db mice as compared with db/+ m mice (Fig. 3(b)). Although 0·1 % Enzamin treatment exerted no effect on insulin sensitivity in db/db mice, 1·0 % Enzamin treatment significantly decreased the plasma glucose level in response to insulin at 30, 60, 90 and 120 min after intraperitoneal insulin injection in db/db mice (Fig. 3(b)). Taken together, these data suggest that Enzamin treatment can ameliorate insulin resistance in db/db mice.

**Fig. 4.**
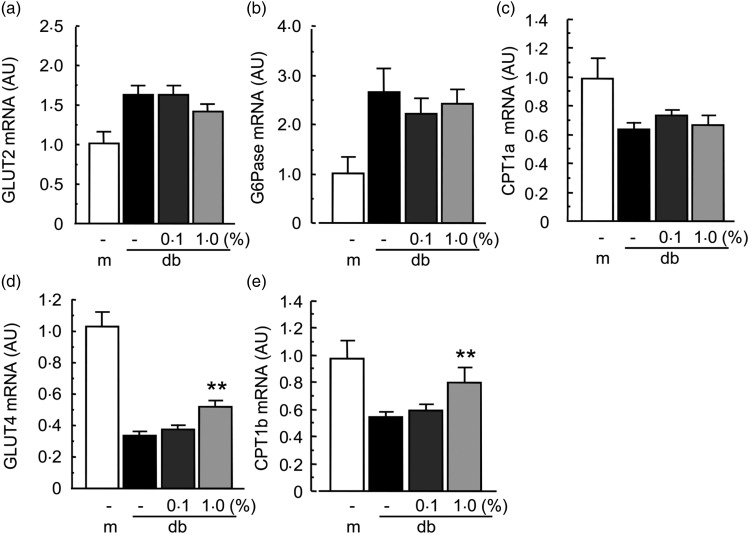
Effect of Enzamin treatment for 8 weeks on glucose and lipid metabolism in liver and muscle. Messenger RNA (mRNA) expression of GLUT2 (a), glucose-6-phosphatase (G6Pase) (b) and carnitine palmitoyltransferase (CPT) 1a (c) in the liver, and GLUT4 (d) and CPT1b (e) in the muscle of non-treated db/ + m (lean control, *n* 6) and db/db (obese control, *n* 10) mice, and 0·1 % (*n* 9) and 1·0 % (*n* 8) Enzamin-treated db/db mice. AU, arbitrary units; m, db/ + m; db, db/db. Data are means (*n* 6–10 per group), with standard errors represented by vertical bars. **Mean value was significantly different from that of the non-treated db/db mice (*P* < 0·01).

We next evaluated the gene expression of GLUT2 (the main GLUT in liver) and glucose-6-phosphatase (G6Pase, an enzyme related to gluconeogenesis) in the liver, and the gene expression of GLUT4 (a muscular GLUT) in the muscle of db/db mice. Enzamin treatment did not affect the mRNA expression of GLUT2 and G6Pase in the liver of db/db mice (Fig. 4(a) and (b)), suggesting that Enzamin does not affect glucose metabolism in the liver of db/db mice. The mRNA expression of GLUT4 in muscle was markedly decreased in db/db mice as compared with db/+ m mice (Fig. 4(d)). Enzamin treatment at 1·0 % significantly increased the mRNA expression of GLUT4 in the muscle of db/db mice as compared with non-treated db/db mice (Fig. 4(d)). We evaluated the gene expression of carnitine palmitoyltransferase 1 (CPT1, a mitochondrial enzyme related with lipid oxidation) in the liver and muscle of db/db mice. The mRNA expression of CPT1a (liver isoform) and CPT1b (muscle isoform) was significantly decreased in db/db mice as compared with db/+ m mice (Fig. 4(c) and (e)). Enzamin treatment at 1·0 % increased the mRNA expression of CPT1b in the muscle of db/db mice as compared with non-treated db/db mice (Fig. 4(e)), whereas Enzamin treatment did not affect the mRNA expression of CPT1a in the liver of db/db mice (Fig. 4(c)). These data suggest that Enzamin treatment improves glucose uptake and lipid oxidation in the muscle of db/db mice.

### Effect of Enzamin on adipocytokine expression in db/db mice

**Fig. 5.**
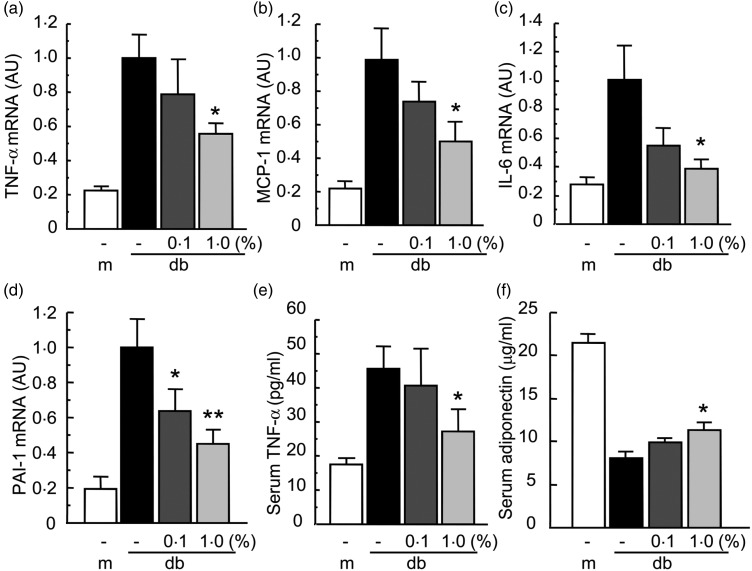
Effect of Enzamin treatment for 8 weeks on adipocytokine expression. Messenger RNA (mRNA) expressions of TNF-α (a), monocyte chemoattractant protein 1 (MCP-1) (b), IL-6 (c) and plasminogen activator inhibitor 1 (PAI-1) (d) in epididymal white adipose tissue, and serum concentrations of TNF-α (e) and adiponectin (f) in non-treated db/ + m (lean control, *n* 6) and db/db (obese control, *n* 10) mice, and 0·1 % (*n* 9) and 1·0 % (*n* 8) Enzamin-treated db/db mice. AU, arbitrary units; m, db/ + m; db, db/db. Data are means (*n* 6–10 per group), with standard errors represented by vertical bars. Mean value was significantly different from that of the non-treated db/db mice: *P < 0·05, **P < 0·01.

To clarify the mechanism whereby Enzamin improves insulin resistance, we examined the effect of Enzamin treatment on pro-inflammatory adipocytokine expression in the adipose tissue of db/db mice. The mRNA expression of TNF-α in the adipose tissue of db/db mice was 5-fold higher than that in db/+ m mice, indicating adipose tissue inflammation in db/db mice (Fig. 5(a)). Enzamin treatment suppressed the TNF-α mRNA expression dose-dependently in the adipose tissue of db/db mice. In particular, 1·0 % Enzamin treatment significantly suppressed TNF-α mRNA expression by 40 % in the adipose tissue of db/db mice (Fig. 5(a)). Consistent with this, 1·0 % Enzamin also suppressed the mRNA expression of MCP-1 and IL-6 by 50 and 60 %, respectively, in the adipose tissue of db/db mice (Fig. 5(b) and (c)). Furthermore, the PAI-1 mRNA level was significantly reduced by both 0·1 and 1·0 % Enzamin in the adipose tissue of db/db mice (Fig. 5(d)). These data suggest that Enzamin treatment can suppress adipose tissue inflammation in db/db mice.

To evaluate the effect of Enzamin on systemic inflammation, we measured the serum TNF-α level in db/db mice. Enzamin treatment at 1·0 %, but not at 0·1 %, significantly suppressed the circulating TNF-α level in db/db mice (Fig. 5(e)), suggesting that Enzamin could suppress systemic inflammation as well as adipose tissue inflammation in db/db mice. In contrast to pro-inflammatory adipocytokines, the level of serum adiponectin was significantly increased by treatment with Enzamin in a dose-dependent manner. In particular, 1·0 % Enzamin treatment increased serum adiponectin by 1·4-fold in db/db mice (Fig. 5(f)). These data suggest that Enzamin can improve the impaired adipocytokine expression in obese mice.

### Effect of Enzamin on macrophage accumulation and activation in db/db mice

**Fig. 6.**
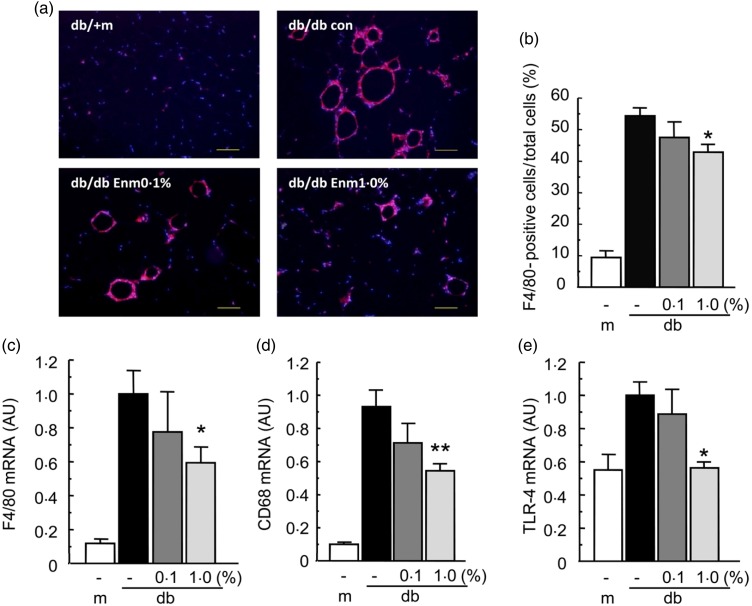
Effect of Enzamin (Enm) treatment for 8 weeks on macrophage accumulation in adipose tissue. (a) Macrophage content of epididymal white adipose tissue (WAT) as assessed by F4/80 staining (bar, 100 µm) and (b) fraction of adipose tissue macrophages (F4/80-stained cells/total cells) in epididymal WAT of non-treated db/ + m (lean control) and db/db (obese control) mice, and 0·1 % and 1·0 % Enzamin-treated db/db mice (*n* 5 for each group). con, Control. Messenger RNA (mRNA) expressions of EGF-like module-containing mucin-like hormone receptor-like 1 (Emr-1) (c), CD68 (d) and Toll-like receptor 4 (TLR-4) (e) in adipose tissue of non-treated db/ + m (lean control, *n* 6) and db/db (obese control, *n* 10) mice, and 0·1 % (*n* 9) and 1·0 % (*n* 8) Enzamin-treated db/db mice. AU, arbitrary units; m, db/ + m; db, db/db. Data are means, with standard errors represented by vertical bars. Mean value was significantly different from that of the non-treated db/db mice: *P < 0·05, **P < 0·01.

To evaluate the effect of Enzamin on macrophage accumulation in adipose tissue, we performed immunohistochemical staining for F4/80, a mature macrophage marker, in adipose tissue sections from obese mice. The histological photographs indicated that macrophage accumulation was dramatically increased in db/db mice as compared with db/+ m mice, and Enzamin treatment appeared to reduce dose-dependently the F4/80-positive area in the adipose tissue of db/db mice (Fig. 6(a)). Consistent with the histological photographs, the fraction of F4/80-positive cells in the adipose tissue was slightly, but not significantly, suppressed by 0·1 % Enzamin, and significantly suppressed by 1·0 % Enzamin treatment in db/db mice (Fig. 6(b)). Furthermore, 1·0 % Enzamin treatment significantly decreased the mRNA expression of EGF-like module-containing mucin-like hormone receptor-like 1 (Emr-1; an F4/80 antigen) in the adipose tissue of db/db mice by 40 % (Fig. 6(c)). In addition, the mRNA expressions of CD68 and Toll-like receptor 4 (TLR-4), a marker of activated and pro-inflammatory macrophages, were decreased by Enzamin treatment in the adipose tissue of db/db mice (Figs. 6(d) and (e)). These data indicate that Enzamin may suppress pro-inflammatory macrophage accumulation and activation in the adipose tissue of db/db mice.

### Effect of Enzamin on oxidative stress in adipose tissue of db/db mice

**Fig. 7.**
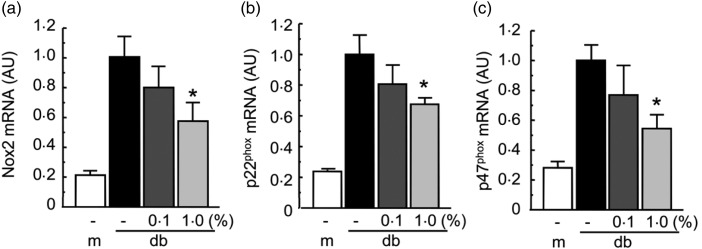
Effect of Enzamin treatment for 8 weeks on oxidative stress in adipose tissue. Messenger RNA (mRNA) expressions of NADPH oxidase (Nox) subunits, Nox2 (a), p22^phox^ (b) and p47^phox^ (c) in adipose tissue of non-treated db/ + m (lean control, *n* 6) and db/db (obese control, *n* 10) mice, and 0·1 % (*n* 9) and 1·0 % (*n* 8) Enzamin-treated db/db mice. AU, arbitrary units; m, db/ + m; db, db/db. Data are means (*n* 6–10 per group), with standard errors represented by vertical bars. *Mean value was significantly different from that of the non-treated db/db mice (*P* < 0·05).

To evaluate the effect of Enzamin on oxidative stress in adipose tissue, we measured the expression of NADPH oxidase subunits in adipose tissue from db/db mice. The expressions of NADPH oxidase subunits, such as NADPH oxidase 2 (Nox2), p22^phox^ and p47^phox^, in non-treated db/db mice were markedly higher than those in db/+ m mice (Fig. 7(a)-(c)). Although there was no significant effect, 0·1 % Enzamin treatment tended to suppress the expressions of NADPH oxidase subunits. Furthermore, treatment with 1·0 % Enzamin significantly suppressed the mRNA expressions of Nox2, p22^phox^ and p47^phox^ by 40, 30 and 40 %, respectively, in the adipose tissue of db/db mice (Fig. 7(a)–(c)). These data suggest that Enzamin treatment can suppress oxidative stress in the adipose tissue of obese mice.

### *Effect of Enzamin on lipopolysaccharide-induced inflammatory response in macrophages* in vitro

**Fig. 8.**
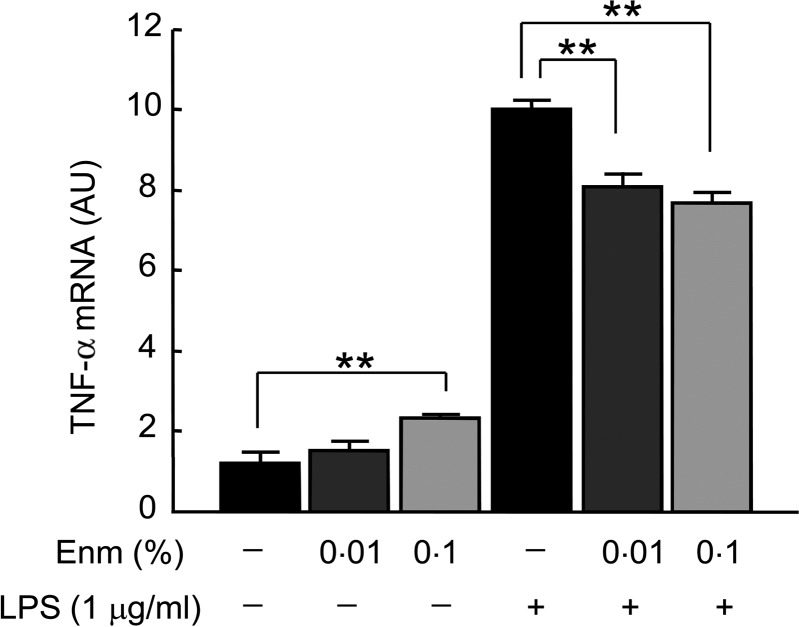
Effect of Enzamin (Enm) treatment on lipopolysaccharide (LPS)-induced inflammatory response in macrophage *in vitro*. Messenger RNA (mRNA) expressions of TNF-α in RAW 264.7 cells treated without or with Enzamin (0·01 and 0·1 %) for 12 h, in the absence or presence of LPS (1 µg/ml). AU, arbitrary units. Data are means (*n* 3 per group), with standard errors represented by vertical bars. **P < 0·01.

To evaluate the influence of Enzamin on the inflammatory response in macrophages, we investigated the effect of Enzamin on LPS-induced TNF-α expression in RAW 264.7 cells, a murine macrophage cell line. Although Enzamin treatment slightly increased the mRNA expression of TNF-α in macrophages without LPS stimulation, the LPS-induced elevation of TNF-α expression was significantly suppressed by both the 0·01 and 0·1 % Enzamin treatments (Fig. 8), suggesting that Enzamin tends to suppress the pro-inflammatory response in macrophages.

## Discussion

In the present study, we demonstrated that Enzamin can improve the insulin resistance and impaired adipocytokine expression in db/db mice. We found that Enzamin treatment suppressed macrophage accumulation in the adipose tissue as well as adipose tissue inflammation in db/db mice. Several studies have shown that the expression of pro-inflammatory cytokines by adipose tissue is in part attributable to the expression of these cytokines by non-adipocytes, including macrophages^(^^12^^,^^13^^)^. Furthermore, Suganami *et al.*^(^^14^^,^^15^^)^ developed an *in vitro* co-culture system composed of adipocytes and macrophages, and demonstrated that a paracrine loop involving NEFA and TNF-α derived from the adipocytes and macrophages, respectively, leads to a vicious cycle that augments the inflammatory changes in both adipocytes and macrophages, i.e. marked up-regulation of pro-inflammatory cytokines such as MCP-1 and TNF-α and down-regulation of the anti-inflammatory cytokine adiponectin. Moreover, in a previous study, we found that pharmacological inhibition of macrophage infiltration suppressed adipose tissue inflammation and improved the impairment of adipocytokine production^(^^16^^)^. In the present experiments, therefore, Enzamin might have suppressed adipose tissue inflammation by inhibiting macrophage infiltration into the adipose tissue of obese mice.

Many reports have suggested that adipose tissue inflammatory changes contribute to the development of insulin resistance. It has been demonstrated that adipose tissue-derived pro-inflammatory cytokines such as TNF-α^(^^17^^,^^18^^)^, and IL-6^(^^19^^,^^20^^)^ can actually cause insulin resistance in experimental models. In the present study, Enzamin treatment at 1·0 % reduced the serum TNF-α level in db/db mice, suggesting that Enzamin can suppress systemic inflammation in db/db mice. Furthermore, TNF-α dose-dependently reduces the expression of adiponectin in adipocytes by suppressing its promoter activity^(^^21^^,^^22^^)^. Adiponectin serves as an insulin-sensitising agent^(^^6^^,^^23^^)^, so that a decrease in plasma adiponectin is related to insulin resistance in obesity. In the present study, we observed that the serum adiponectin level was decreased in db/db mice as compared with db/ + m mice, in addition to an impaired insulin sensitivity and glucose tolerance. However, Enzamin treatment improved hypoadiponectinaemia in db/db mice. The increase in adiponectin by Enzamin treatment therefore may be responsible for the improvement of insulin resistance in db/db mice. Furthermore, we also found that Enzamin treatment increased the expression of GLUT4 mRNA, which is associated with glucose uptake in muscle of db/db mice. Previous reports suggest that the impairment of insulin signalling through the reduction of GLUT4 expression and translocation in muscle is responsible for the insulin resistance in db/db mice^(^^24^^,^^25^^)^. It has been shown that TNF-α impairs insulin receptor signalling through the impairment of GLUT4 expression and translocation to the plasma membrane^(^^26^^)^. Therefore, Enzamin might improve glucose uptake in the muscle of db/db mice, presumably through the reduction of circulating TNF-α.

Enzamin treatment could also suppress the gene expression of the NADPH oxidase subunit, which is associated with oxidative stress, in the adipose tissue of db/db mice. In addition to inflammation, oxidative stress plays a critical role in insulin resistance^(^^27^^)^. In fact, antioxidants such as vitamins C and E, and α-lipoic acid ameliorate insulin resistance^(^^28^^,^^29^^)^. Oxidative stress is known to be increased in obesity via NADPH oxidase activation^(^^30^^,^^31^^)^. NADPH oxidase is a major source of reactive oxygen species in various organs, especially in white adipose tissue^(^^30^^,^^31^^)^. NADPH oxidase consists of the membrane-associated flavocytochrome b558 family of proteins, which include Nox2 (gp91^phox^) and p22^phox^, as well as the cytosolic components p47^phox^, p67^phox^ and p40^phox^^(^^32^^)^. In the present study, we observed that Enzamin treatment suppressed the gene expressions of these subunits. Our findings suggest that an antioxidative effect of Enzamin may also be involved in the improvement of insulin resistance.

We found that Enzamin treatment suppressed LPS-induced TNF-α expression in macrophages *in vitro*, indicating that Enzamin can directly suppress the pro-inflammatory response in macrophages. Furthermore, we observed that Enzamin treatment suppressed activated macrophage markers, such as CD68 and Toll-like receptor 4 (TLR-4), in the adipose tissue of db/db mice. These findings suggest that Enzamin may suppress the inflammatory response in macrophages.

Morange *et al.*^(^^33^^)^ reported that PAI-1 deficiency improved insulin resistance and markedly increased tissue-type plasminogen activator (t-PA) activity, but not urokinase-type plasminogen activator (u-PA) activity in the adipose tissue of obese mice. Furthermore, the levels of plasma insulin and blood glucose in obese t-PA-deficient mice were higher than those in obese wild-type (WT) mice, suggesting that t-PA is associated with insulin sensitivity^(^^34^^)^. We observed that Enzamin treatment markedly decreased the levels of PAI-1 mRNA in the adipose tissue of db/db mice. Furthermore, we previously demonstrated that Enzamin treatment increased t-PA activity, but not u-PA activity, in the blood of mice. We also reported that Enzamin contains the substances that strongly bind t-PA assessed by the IAsys resonance assay^(^^9^^)^. Therefore, it is assumed that the pro-fibrinolytic state induced by Enzamin might be associated with the improvement of insulin resistance in obese mice. However, further study will be required to clarify the mechanisms by which Enzamin improves insulin resistance in db/db mice.

In conclusion, the present data suggest that Enzamin can ameliorate insulin resistance presumably through suppression of the inflammatory response and oxidative stress in adipose tissue. Since Enzamin has been utilised in health care and no side effects have been reported, it may represent a beneficial supplement for the prevention of the metabolic syndrome.
